# Scleroderma clinical trials consortium classification criteria for systemic sclerosis heart involvement

**DOI:** 10.1093/rheumatology/keag109

**Published:** 2026-02-27

**Authors:** Laura Ross, Andrew T Burns, André La Gerche, Dylan Hansen, J Gerry Coghlan, Wendy Stevens, David Prior, Alan Pham, Penny McKelvie, Chiara Bellocchi, Yolanda Braun Moscovici, Cosimo Bruni, Patricia Carreira, Tracy Frech, Sabrina Hoa, Marie Hudson, Vivien Hsu, Andrea Hsiu Ling Low, Marco Matucci-Cerinic, Benjamin Medina Fonseca, Sue-Ann Ng, Tatiana Rodriguez Reyna, Joanne Sahhar, Mohamed Talaat, Susanna Proudman, Alessandra Vacca, Murray Baron, Mandana Nikpour, and the SCTC Cardiac Working Group, Catherine Abric, Monica Alouian, Shervin Assassi, Murray Baron, Cosimo Bruni, Maya Buch, Andreea Bujor, Andrew Burns, Lorinda Chung, Benedict Costello, M E Csuka, Francesco Del Galdo, Christopher P Denton, Jeska de Vries-Bouwstra, Girish Dwivedi, Tracy Frech, J Gerry Coghlan, Clive Handler, Ariane Herrick, Monique Hinchcliff, Alicia Hinze, Sabrina Hoa, Vivien Hsu, Marie Hudson, Laura Hummers, Jacqueline Joza, Kusano Kengo, Dinesh Khanna, André La Gerche, David Langleben, Thomas Medsger, Edward Miller, Roberta Montisci, Mandana Nikpour, Gene-Siew Ngian, John D Pauling, David Prior, Susanna Proudman, Tatiana Rodrigeuz-Reyna, Lawrence Rudski, Joanne Sahhar, Flora Sam, James Seibold, Virginia Steen, Wendy Stevens, Alessandra Vacca, Jaap van Laar, Alison Hendry

**Affiliations:** Department of Medicine, University of Melbourne, Parkville, VIC, Australia; Department of Rheumatology, St Vincent’s Hospital Melbourne, Fitzroy, VIC, Australia; Department of Medicine, University of Melbourne, Parkville, VIC, Australia; Department of Cardiology, St Vincent’s Hospital Melbourne, Fitzroy, VIC, Australia; Department of Cardiology, St Vincent’s Hospital Melbourne, Fitzroy, VIC, Australia; Heart, Exercise and Research Trials (HEART) Lab, St Vincent's Institute, Fitzroy, VIC, Australia; Heart, Exercise and Trials (HEART) Lab, Victor Chang Cardiac Research Institute, Darlinghurst, NSW, Australia; Department of Rheumatology, St Vincent’s Hospital Melbourne, Fitzroy, VIC, Australia; Department of Cardiology, Royal Free Hospital, London, UK; Institute of Cardiovascular Sciences, University College London, London, UK; Department of Medicine, University of Melbourne, Parkville, VIC, Australia; Department of Rheumatology, St Vincent’s Hospital Melbourne, Fitzroy, VIC, Australia; Department of Medicine, University of Melbourne, Parkville, VIC, Australia; Department of Anatomical Pathology, The Alfred Hospital, Melbourne, VIC, Australia; Department of Anatomical Pathology, St Vincent’s Hospital Melbourne, Fitzroy, VIC, Australia; Department of Clinical Sciences and Community Health, Dipartimento di Eccellenza, University of Milan, Milan, Italy; Referral Center for Systemic Autoimmune Diseases, Foundazione IRCCS Ca’Granda Osepdale Maggiore Policlinico di Milano, Milan, Italy; Rheumatology Institute, Rambam Health Care Campus, Rappaport Faculty of Medicine, Technion-Israeli Institute of Technology, Haifa, Israel; Department of Experimental and Clinical Medicine, University of Florence, Florence, Italy; Rheumatology Department, University Hospital 12 de Octubre, Madrid, Spain; Department of Medicine, Vanderbilt University Medical Center, Nashville, TN, USA; Division of Rheumatology and Immunology, Tennessee Valley Healthcare System, Nashville, TN, USA; Department of Medicine Centre Hospitalier de l’Université de Montréal, Montreal, Quebec, Canada; Division of Rheumatology, Jewish General Hospital, Montreal, Quebec, Canada; Department of Medicine, McGill University, Montreal, Quebec, Canada; Department of Medicine, Rutgers-Robert Wood Johnson Medical School, Rutgers University, New Brunswick, NJ, USA; Department of Rheumatology and Immunology, Singapore General Hospital, Singapore; Department of Rheumatology, Duke-NUS, National University of Singapore, Singapore; Department of Experimental and Clinical Medicine, University of Florence, Florence, Italy; Department of Rheumatology and Internal Medicine, Instituto Nacional de Ciencias Médicas y Nutrición Salvador Zubirán, Mexico City, Mexico; Department of Rheumatology and Immunology, Singapore General Hospital, Singapore; Department of Rheumatology, Duke-NUS, National University of Singapore, Singapore; Department of Rheumatology and Internal Medicine, Instituto Nacional de Ciencias Médicas y Nutrición Salvador Zubirán, Mexico City, Mexico; Department of Medicine, Monash University, Clayton, VIC, Australia; Department of Rheumatology, Monash Health, Clayton, VIC, Australia; Department of Medicine, Rutgers-Robert Wood Johnson Medical School, Rutgers University, New Brunswick, NJ, USA; Rheumatology Unit, Royal Adelaide Hospital, Adelaide, SA, Australia; Discipline of Medicine, University of Adelaide, Adelaide, SA, Australia; Rheumatology Unit, University of A.O.U. of Cagliari, Via Università, Cagliari, Italy; Division of Rheumatology, Jewish General Hospital, Montreal, Quebec, Canada; School of Public Health, University of Sydney, Sydney, NSW, Australia; Department of Rheumatology, Royal Prince Alfred Hospital, Camperdown, NSW, Australia; Sydney MSK Research Centre, University of Sydney, Sydney, NSW, Australia

**Keywords:** systemic sclerosis, classification criteria, cardiac, myocardial

## Abstract

**Objectives:**

Systemic sclerosis-associated heart involvement (SHI) is an enigmatic disease manifestation associated with high mortality. The Scleroderma Clinical Trials Consortium (SCTC) Cardiac Working Group developed SHI classification criteria to enable systematic investigation of this condition.

**Methods:**

An international, interdisciplinary working group was assembled. Using consensus methods and existing literature, provisional SHI classification criteria items were developed. Continuous consensus exercises and a discrete choice experiment were performed to reduce items and derive individual item weights. The sensitivity and specificity of the classification criteria were tested in an independent cohort (*n* = 168) of SHI (cases) and non-SSc heart disease (controls).

**Results:**

The working group agreed that the SCTC SHI Classification Criteria should identify the direct effects of SSc on the heart and exclude the complications of other SSc manifestations or cardiac comorbidities. The final classification criteria include 23 items measuring cardiac fibrosis, inflammation, arrhythmias and small vessel vasculopathy. No single item is pathognomonic for SHI, with a requirement for the presence of abnormalities across multiple histopathological, imaging, serological, and imaging domains to be present to secure a diagnosis. A classification criteria score of ≥11 identified SHI with a sensitivity of 78% and specificity of 96%, with an area under the curve of 0.87 (0.80–0.93). This threshold correctly identified >90% of cases of SHI.

**Conclusion:**

The newly derived SCTC SHI Classification Criteria have high sensitivity and specificity for SHI. Application of these criteria will enable standardized classification of patients in studies to facilitate future investigation of this important disease manifestation.

## Graphical abstract

**Graphical Abstract:**
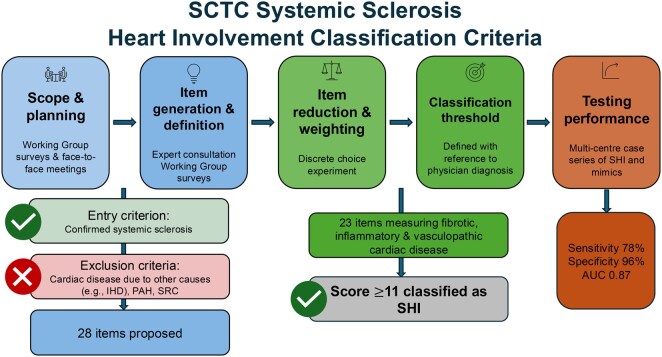

Rheumatology key messages

- This study presents the first classification criteria for the classification of SSc heart involvement.
- A score of ≥11 identifies SSc heart involvement with high sensitivity and specificity.
- Standardized criteria will enable future studies to identify biomarkers for heart involvement and therapeutic studies to identify effective treatments.

## Introduction

SSc can affect all major organ systems, including the heart, with a spectrum of cardiac disease ranging from florid myocarditis to asymptomatic chronic pericardial effusion [1]. Studies since the 1940s have reported the direct effects of SSc on the heart [2], and autopsy studies document heart involvement in up to 90% of cases [3–5]. SSc-associated heart involvement (SHI) remains a leading cause of mortality, with no disease-modifying therapy available [1, 6, 7].

A consensus definition of SHI has been endorsed by the World Scleroderma Foundation (WSF) and Heart Failure Association of the European Society of Cardiology (HFA), stating that SHI is defined by the presence of ‘cardiac abnormalities predominantly attributable to SSc rather than other causes and/or complications. SHI may be subclinical and must be confirmed through diagnostic investigation. The pathogenesis of SHI comprises one or more of inflammation, fibrosis and vasculopathy’ [8]. There are no validated criteria by which to classify SHI. Highly variable case ascertainment means SHI is variably quantified, coupled with increasing evidence demonstrating the importance of subtle changes of cardiac structure and function [9–12]. The natural history of SHI is poorly defined and there are limited methods to identify high-risk patients [1].

The Scleroderma Clinical Trials Consortium (SCTC) Cardiac Working Group (WG) sought to develop SHI classification criteria. These criteria would enable accurate identification and quantification of SHI, and risk and prognostic factors for SHI, and facilitate therapeutic trials.

## Methods

### Investigators

**Figure 1:**
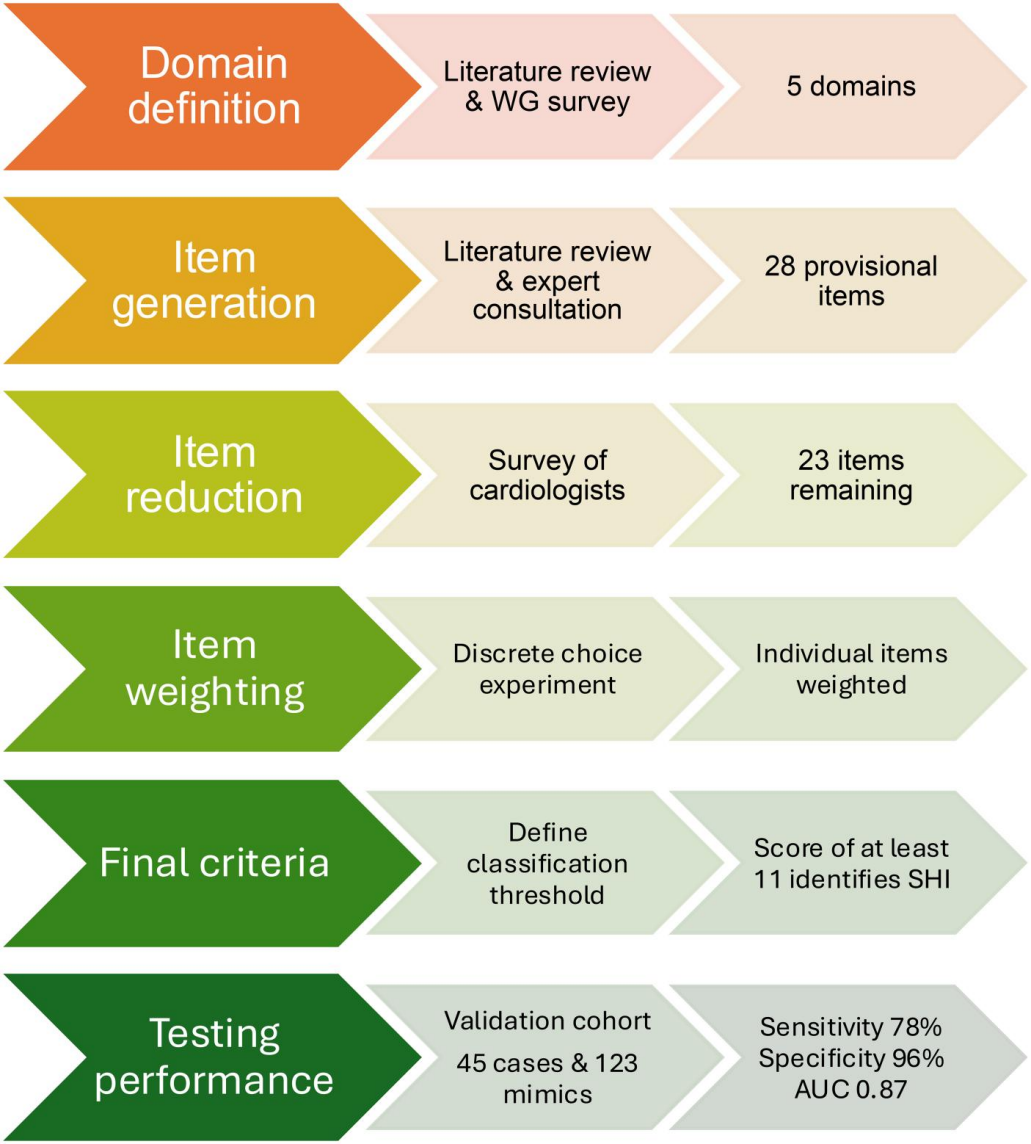
Development of the Scleroderma Clinical Trials Consortium SSc heart involvement classification criteria. AUC: area under the curve; WG: working group

The SCTC Cardiac WG included 24 rheumatologists, 5 cardiologists and 2 anatomical pathologists. The ACR propose methodological guidance for the development of classification criteria (https://rheumatology.org/criteria). These methods were adapted to develop these criteria (Fig. 1).

### Domain definition

A scoping literature review was performed to identify previous definitions of SHI and generate potential criteria items [1]. Using these results, a survey to define the domains of disease to be included in the classification criteria was developed. WG members completed a single-round survey, with results presented at an in-person meeting. An *a priori* threshold of 70% agreement for inclusion or exclusion of a domain was set.

### Expert consultation

Rheumatologists (*n* = 4), cardiologists (*n* = 5) and anatomical pathologists (*n* = 2) were consulted to generate provisional classification criteria items. Cardiologists had expertise in cardiac imaging [echocardiography and cardiac MRI (CMR)], heart failure and pulmonary vascular disease. Anatomical pathologists had expertise in cardiac and skeletal muscle histopathology. The expert panel was presented with literature review and WG survey results, and asked to suggest items to measure each domain. In an iterative process, experts were asked to provide feedback on the items suggested by other expert panel members. Disagreement about proposed items was resolved by consensus. A face-to-face meeting including two cardiologists and two rheumatologists from the expert panel reviewed all suggested items, and provisional items were grouped according to the underlying SHI pathophysiological mechanisms: myocarditis, myocardial fibrosis, vascular abnormalities and pericardial abnormalities. All preliminary items were distributed to expert panel members to confirm agreement with the provisional items.

### Item reduction

Fifteen cardiologists from Europe, the UK, North America and Asia-Pacific, with experience in the assessment and management of heart disease due to SSc and other autoimmune diseases, were invited to participate in an online survey. Respondents were asked to rate the specificity of each provisional SHI criterion on a Likert scale from 1 = highly unlikely to indicate SHI to 7 = highly likely to indicate SHI. Items that were rated as at least somewhat likely (score ≥5) by at least 50% of experts were retained. Survey results were presented to all participating cardiologists to assess the face and content validity of the proposed list of classification items.

### Item weighting

Cardiologists and rheumatologists were invited to participate in an online discrete choice experiment (DCE) to assign weights to individual items using multicriteria decision analysis [13]. Respondents were presented with a series of paired scenarios and asked to rate which of the scenarios was most likely a case of SHI. The DCE was hosted on the 1000minds platform (http://www.1000minds.com). The PAPRIKA (Potentially All Pairwise RanKings of all possible Alternatives) method was applied to derive a relative ranking for each criterion [13]. Item weights were calculated by rounding the relative item weights to the nearest integer.

### Defining a classification threshold

Twenty cases, representing a range of SHI presentations and heart disease mimickers, were collected to enable definition of a SHI classification threshold. Cases were selected to include the spectrum of features reported to indicate the presence of SHI [1]. These cases were presented to cardiologists and rheumatologists in a standardized format.

All cardiologists who completed the item reduction survey and 22 rheumatologists from Europe, the UK, North and Central America and Asia-Pacific were asked to rate their diagnostic certainty about each case on a numerical rating scale from 0 to 20, where 0 = definitely not SHI to 20 = definitely SHI. Respondents were not provided with data from previous phases of the criteria development and were blinded to other participants’ rankings. Median scores for each case were calculated by pooling all responses.

Physician determined presence of SHI was the gold standard comparison to define the SHI classification threshold. This is consistent with the current SHI diagnostic standard of physician diagnosis [8]. This approach has been applied when developing classification criteria for other systemic autoimmune diseases [14].

Analysis of case ranking scores showed a score of 15 was the threshold above which a case was ranked in the top quartile of cases most likely to be SHI. This was defined as the threshold of highly likely or definite SHI. This threshold was set to optimize the specificity of the classification criteria given their intended purpose for use as a research tool. Cases that were ranked by >50% of experts as highly likely SHI (score ≥15) were considered positive for SHI.

Each of the 20 cases was assigned a SHI classification criteria score by summing the individual item weights derived from the DCE. Cases that scored around the provisional classification threshold were reviewed again by two expert rheumatologists and one cardiologist to ascertain which cases could be confidently classified as having SHI. A threshold for the classification of SHI was identified based on targets of >90% specificity and >80% sensitivity.

### Testing the performance of the classification criteria

SCTC members were invited to submit cases of SHI and non-SSc associated heart disease, to form a cohort of cases in which to test the new criteria. Cases of non-SHI were collected to form a control group. Submitted cases could be either historical or contemporary. No case used to define the classification threshold was included in this cohort. Cases were all submitted prior the definition of any classification thresholds for SHI. Submitting members did not participate in the item generation or reduction phases of the criteria development.

Data were entered into a standardized data collection form that captured clinical symptoms, examination findings, and electrocardiographic, imaging and histopathological findings. A complete set of investigations were not required for a case to be included, as the goal was to collect a real-world cohort of SHI cases reflective of contributing members’ clinical experience. Contributing members were asked to nominate whether their patient had SHI or heart disease of another aetiology, using a Likert scale that ranged from 1 = definitely SHI to 7 = definitely not SHI. This was the reference gold standard test for the presence of SHI. Physician ratings of cases were reviewed by two authors (L.R., M.B.) to ensure consistency of rating of cases as confirmed SHI or not SHI before any calculation of classification criteria scoring. The clinical characteristics of those with and without SHI in this cohort were compared using chi-squared or Fisher’s exact test as appropriate for discrete variables and Wilcoxon rank-sum test for non-normally distributed continuous variables. A SHI classification criteria score was calculated for each case. Cases that were positive for any of the SHI classification exclusion criteria were scored as 0. Any missing items were scored as 0, i.e. if a case had no CMR results, the case would score zero points for any CMR classification criteria items. This approach was chosen to avoid imputing unmeasured disease features based upon assumptions about SSc cardiac disease.

A case was considered positive for SHI if a case was rated as 1 = definitely SHI or 2 = highly likely SHI. All other cases of uncertain SHI or non-SSc heart disease were considered as negative for SHI. Performance of the classification criteria was assessed using a preliminary threshold of ≥11 points. This threshold was selected based on sensitivity and specificity testing. Further sensitivity analyses of alternate classification thresholds were tested. Sensitivity and specificity were calculated, with discrimination tested by calculating the area under the curve (AUC) of receiver operator characteristic curves. Logistic regression analysis was performed to calculate the odds ratio of a specific SHI classification criteria threshold detecting SHI. The percentage of correctly classified cases was recorded.

Study data were collected and managed using REDCap electronic data capture tools hosted at The University of Melbourne. All statistical analyses, excluding analysis of the DCE, were performed using STATA 18.0 (StataCorp, College Station, TX, USA). Ethics approval for this study was granted by the Human Research Ethics Committee at St Vincent’s Hospital Melbourne (LRR 195/20).

## Results

### Domain generation

The WG reached consensus to develop SHI classification criteria that could be applied to clinical trials and observational studies. The group agreed that criteria should focus on primary heart involvement and not include cardiac complications of other SSc manifestations such as pulmonary arterial hypertension (PAH), interstitial lung disease or scleroderma renal crisis (SRC). It was agreed that SHI classification criteria should distinguish the direct effects of SSc on the heart from other cardiac comorbidities, consistent with the WSF/HFA definition of SHI [8].

Twenty-two WG members (15 rheumatologists, 7 cardiologists) were invited to participate in the initial domain-defining survey. Sixteen (73% response rate; 11/15 rheumatologists, 5/7 cardiologists) complete responses were received. WG members were asked to identify domains of cardiac disease that could identify SHI in a clinical trial. The following domains of cardiac disease were identified for inclusion: myocardial fibrosis, myocardial inflammation, arrhythmias, conduction abnormalities, pericardial abnormalities. Sixty-nine percent of respondents agreed perfusion abnormalities should be a domain. Valvular heart disease was omitted at this stage, as 80% of respondents agreed that it could not be used to identify SHI in clinical trials.

### Item generation

With consideration to the scoping literature review [1] and domain-generation survey results, the expert panel iteratively generated potential items to measure the proposed SHI domains. The expert panel agreed that arrhythmias and conduction abnormalities are clinical manifestations of underlying myocardial fibrosis or inflammation, so items pertaining to these domains were grouped within the fibrosis and inflammation domains. Vascular abnormalities that may result from SHI were considered, given this domain almost reached the threshold of agreement. The expert panel agreed that microvasculopathy could be consistent with SHI, therefore, a vascular domain was retained.

In recognition of the fact that changes of SHI can mimic several other conditions, the expert panel agreed to have exclusion criteria as part of the SHI classification criteria. The following exclusion criteria were developed: (i) a diagnosis of SHI can only be made in the setting of an established diagnosis of SSc; and (ii) SHI can only be diagnosed when other causes of cardiomyopathy, particularly infiltrative diseases, have been reasonably excluded. Other causes of cardiomyopathy to exclude are cardiac disease secondary to Group I PAH, ischaemic heart disease, hypertensive heart disease, cardiac disease secondary to renal disease, dilated cardiomyopathy (e.g. genetic, post-non-SSc myocarditis, alcohol), cardiac sarcoidosis, cardiac amyloidosis, iron overload and Fabry’s disease. The expert group generated 28 items to measure SHI across four domains of cardiac disease: myocardial fibrosis, myocardial inflammation, vascular abnormalities and pericardial abnormalities.

### Item reduction and weighting

Fifteen cardiologists were invited to complete the item reduction survey. Cardiologists were asked to consider the specificity of each of the preliminary criteria for SHI. Twelve (80%) complete responses were received. Three items (left ventricular hypertrophy, atrial fibrillation, cardiac tamponade) were omitted in this phase as they did not meet the *a priori* threshold of agreement, indicating inadequate specificity for SHI. Reduced ventricular ejection fraction and ventricular dysfunction were combined into a single item. Twenty-one rheumatology and cardiology experts participated in an online DCE. A relative ranking and item weight were generated for each criterion. The final SHI classification criteria items are listed in Table 1.

**Table 1:** Scleroderma Clinical Trials Consortium SHI Classification Criteria.

A diagnosis of SHI can only be made in the setting of an established diagnosis of SSc SHI can only be diagnosed when other causes of cardiomyopathy, particularly infiltrative diseases, have been reasonably excluded. Other causes of cardiomyopathy to be excluded include: Cardiac disease secondary to Group I pulmonary arterial hypertension Ischaemic heart disease Hypertensive heart disease Cardiac disease secondary to renal disease Dilated cardiomyopathy (e.g. genetic, post-non-SSc myocarditis, alcohol, etc.) Cardiac sarcoidosis, cardiac amyloidosis, iron overload, Fabry’s disease

Classification score ≥11 indicates presence of SHI	
Classification criteria item	Item weight
Endomyocardial and pericardial biopsy	
Perivascular and/or interstitial mononuclear inflammatory infiltrate adjacent to areas of myocyte necrosis and/or degeneration, with or without myocardial fibrosis	6
Perivascular and/or interstitial inflammatory infiltrate in the absence of myocyte necrosis	5
Excess interstitial fibrosis with or without replacement fibrosis	6
Fibrous or fibrinous pericarditis or pericardial adhesions	5
CMR	
Myocarditis as per Lake Louise Criteria	5
Abnormalities on T2 sequences (including T2 mapping) or post-contrast imaging suggestive of myocardial oedema that do not meet Lake Louise criteria for diagnosis of myocarditis	5
Epicardial or mid-wall late gadolinium enhancement in a non-ischaemic pattern	6
Abnormal T1 mapping time	5
Focal thinning or akinesis of myocardial wall or wall motion abnormalities	4
Evidence of thickened pericardium on either CMR, cardiac CT, TTE	4
Objective evidence of myocardial ischaemia in the absence of epicardial coronary artery disease (e.g. transmural scarring on CMR and patent coronary arteries)	4
Echocardiography	
Acute or subacute onset global or regional left and/or right ventricular dysfunction and/or reduced ventricular ejection fraction	4
Grade II or Grade III left ventricular diastolic dysfunction, according to ASE/EACVI criteria	4
Chronic non-trivial pericardial effusion	3
Electrocardiography	
Unexplained 2nd degree Mobitz type II conduction defect or complete heart block	4
Multifocal or complex ventricular ectopics	4
Sustained or non-sustained ventricular tachyarrhythmia	5
Right heart catheterization	
Unexplained post–capillary wedge pressure >15 mmHg or left ventricular end diastolic pressure >12 mmHg	4
Serum biomarkers	
Elevated troponin (above upper limit of normal)	3
Elevated serum BNP/NT-proBNP (above upper limit of normal)	3
Clinical features	
New-onset heart failure	3
Signs, symptoms, investigation findings of constrictive pericarditis^a^	3
Acute pericarditis indicated by clinical symptoms and either typical ECG changes or imaging evidence of pericardial inflammation	4

Patients do not have to complete all investigations to secure a diagnosis of SHI. If a particular investigation has not been performed or the described abnormality is not observed, the patient scores zero for that item.

a Evidence of constrictive pericarditis includes ventricular septal shift, medial mitral e′ and elevated hepatic vein expiratory diastolic reversal ratio measured by echocardiography coupled with clinical signs of heart failure.

ASE: American Society of Echocardiography; BNP: B-type natriuretic peptide; CMR: cardiac MRI; EACVI: European Association of Cardiovascular Imaging; NT-proBNP: N-terminal pro B-type natriuretic peptide; SHI: SSc-associated heart involvement; TTE: transthoracic echocardiography.

### Defining the classification threshold

Twenty-eight survey responses were received (82% response rate). Inspection of case rankings revealed a significant spread of results across the Likert scale (Supplementary Table S1). Seven (35%) cases had >50% of respondents rate the case with a score ≥15. These cases were considered as positive for SHI. Eleven (55%) cases had a median ranking of >10, equating to possible or likely SHI. A SHI classification score was calculated for each case by summing individual item weights. A preliminary classification threshold of ≥11 points identified SHI cases (as per physician diagnosis) with a sensitivity of 100% (95% CI 66.37–100%) and specificity of 81.82% (95% CI 48.22–97.72%).

Table 2 describes the assembled test cohort. A total of 168 cases were submitted of which 45 (27%) were considered to have definite SHI. The median disease duration at time of diagnosis of heart disease was 4 (2–12) years. The median disease duration at time of onset of SHI was 3 (1–8) years, compared with 5 (2–13) years duration in non-SHI cases (*P* = 0.04). The median number of cardiac investigations performed in those classified as having SHI was 5 (4–6), compared with a median number of tests in the non-SHI group of 4 (3–5) (*P* < 0.01). All participants had an echocardiogram.

**Table 2:** Clinical characteristics of the validation cohort.

**Clinical variable**	**SHI** ^b^ **(*n* = 45)**	Not SHI (*n* = 123)	*P*-value
Female (*n*, %)	37 (82.22)	100 (81.30)	0.90
Age at SSc diagnosis (years, median IQR)	33 (22–44)	50 (41–59)	<0.01
Age at onset of heart disease (years, median IQR)	38 (30–55)	60 (50–68)	<0.01
dcSSc (*n*, %)	36 (80)	58 (47.15)	<0.01
Centromere positive (*n*, %)	6 (13.33)	35 (28.46)	0.05
Scl70 positive (*n*, %)	25 (55.56)	42 (34.15)	0.01
RNA polymerase III positive (*n*, %)	0 (0)	11 (8.94)	0.04
U1RNP positive (*n*, %)	3 (6.67)	13 (10.57)	0.56
Disease manifestations prior to onset of heart disease^a^ (*n*, %)			
Digital ulcers	30 (66.67)	63 (51.22)	0.07
Tendon friction rubs	9 (20.00)	14 (11.38)	0.20
Arthritis	18 (40.00)	41 (33.33)	0.42
Interstitial lung disease	27 (60.00)	68 (55.28)	0.59
Myositis	17 (37.78)	21 (17.07)	0.01
Pulmonary arterial hypertension	0 (0.00)	37 (30.08)	<0.01
Scleroderma renal crisis	0 (0.00)	8 (6.50)	0.08
Comorbidities (*n*, %)			
Diabetes mellitus	0 (0.00)	8 (6.50)	0.11
Obesity	1 (2.22)	18 (14.63)	0.03
Hypertension	4 (8.89)	51 (41.46)	<0.01
Stroke	0 (0.00)	1 (0.81)	1.00
Peripheral vascular disease	6 (13.33)	8 (6.50)	0.21
Venous thromboembolic disease	1 (2.22)	8 (6.50)	0.45
Atrial fibrillation	2 (4.44)	15 (12.0)	0.25
Chronic kidney disease	0 (0.00)	15 (12.20)	0.01
Smoker at onset heart disease	4 (8.89)	6 (4.88)	0.46
Symptoms at time of presentation of heart disease (*n*, %)			
Palpitations	21 (46.67)	28 (22.76)	<0.01
Chest pain	5 (11.11)	20 (16.26)	0.41
Acute heart failure	4 (8.89)	11 (8.94)	1.00
Acute pericarditis	2 (4.44)	2 (1.63)	0.29
Progressive skin disease	14 (31.11)	23 (18.70)	0.09
Progressive interstitial lung disease	9 (20.00)	12 (9.76)	0.11
Active myositis	9 (20.00)	8 (6.50)	0.02
Arthritis	6 (13.33)	14 (11.38)	0.79
Tendon friction rub	5 (11.11)	6 (4.88)	0.17
Cardiac investigations (*n*, %)			
ECG performed	33 (73.33)	94 (76.42)	0.68
Non-sinus rhythm	4 (12.12)	12 (13.19)	0.73
Conduction defect	10 (30.30)	22 (31.88)	0.87
24-h Holter performed (*n*, %)	29 (64.44)	34 (27.64)	<0.01
Ventricular ectopy detected	22 (75.86)	24 (77.42)	1.00
Multifocal ventricular ectopy	10 (45.45)	2 (9.52)	0.03
Ventricular tachycardia	7 (25.93)	0 (0.00)	<0.01
Transthoracic echocardiogram performed	45 (100)	123 (100)	
LVEF (%, median, IQR)	60 (49–65)	60 (55–65)	0.22
LV wall motion abnormalities (*n*, %)	15 (34.09)	17 (14.17)	0.01
Impaired RV systolic function (*n*, %)	21 (40.00)	35 (19.27)	0.03
Pericardial effusion (*n*, %)	14 (31.11)	26 (21.49)	0.28
Thickened pericardium (*n*, %)	1 (2.27)	0 (0.00)	0.35
CMR performed	36 (80.00)	27 (21.95)	<0.01
LVEF (%, median, IQR)	57 (49–63)	59 (51–63)	0.54
RVEF (%, median, IQR)	50 (34–56)	54 (40–64)	0.09
LV wall thinning (*n*, %)	4 (11.43)	1 (3.70)	0.42
RV wall thinning (*n*, %)	0 (0.00)	1 (3.70)	0.68
Ventricular wall motion abnormalities (*n*, %)	13 (37.14)	6 (22.22)	0.31
Late gadolinium enhancement (*n*, %)	28 (80.00)	10 (37.04)	<0.01
Elevated T1 mapping time (*n*, %)	6 (13.33)	6 (4.88)	0.07
Abnormal T2 signal (*n*, %)	8 (24.24)	3 (11.54)	0.50
Pericardial effusion (*n*, %)	14 (40.00)	9 (33.33)	0.83
Thickened pericardium (*n*, %)	1 (2.94)	1 (3.70)	0.70
SPECT imaging performed (*n*, %)	0 (0.00)	5 (4.10)	0.33
Right heart catheterization performed (*n*, %)	12 (26.67)	58 (47.15)	0.02
Mean pulmonary artery pressure (mmHg, median, IQR)	21 (20–30)	27 (24–35)	0.04
Pulmonary capillary wedge pressure (mmHg, median, IQR)	13 (8–16)	13 (9–17)	0.83
Pulmonary vascular resistance (Wood units, median, IQR)	2.31 (1.62–3.20)	2.50 (1.75–4.29)	0.43
LV end diastolic pressure (mmHg, median, IQR)	24 (15–25)	16 (11–18)	0.06
Coronary angiogram performed (*n*, %)	10 (22.22)	44 (35.77)	0.14
Coronary artery disease (*n*, %)	0 (0.00)	21 (48.84)	<0.01
Endomyocardial biopsy performed^c^ (*n*, %)	1 (2.22)	1 (0.81)	0.47
Interstitial fibrosis (*n*, %)	1 (100)	1 (100)	
Myocyte degeneration (*n*, %)	1 (100)	0 (0)	

Demographic and clinical data were available for all patients. The number of patients who underwent each cardiac investigation is reported in the table. Frequency of abnormalities of each cardiac test are reported as the percentage of those for whom results were available.

a Clinical manifestations reported by submitting physician.

b SHI determined by classification criteria score ≥11.

c No submitted case reported inflammation or pericardial changes on endomyocardial biopsy.

CMR: cardiac MRI; IQR: interquartile range; LV: left ventricle; LVEF: left ventricular ejection fraction; RV: right ventricle; RVEF: right ventricular ejection fraction; SPECT: single-photon emission computed tomography; Scl70: anti-topoisomerase I; SHI: SSc-associated heart involvement; TTE: transthoracic echocardiography.

The median SHI classification score of the test cohort was 3 (IQR 0–10). Forty cases (24%) had a SHI criteria score ≥11 points. A classification criteria threshold of ≥11 points correctly classified 91.07% of the test cohort with a sensitivity and specificity of 77.78% and 95.93%, respectively. This threshold had good discrimination with an AUC of 0.87 (0.80–0.93) and a diagnostic odds ratio of 82.60 (95% CI 26.47–257.72, *P* < 0.01). The sensitivity and specificity of other thresholds were tested (Supplementary Table S2). Owing to the emphasis placed on specificity for the development of classification criteria, a threshold of ≥11 was considered the most appropriate threshold as this cut point had an excellent AUC, high specificity and high odds ratio for SHI diagnosis.

## Discussion

Following a formal iterative process of testing, we propose the first classification criteria for SHI. Previous work has developed a definition of SHI [8], and these are clinical parameters that can operationalize this definition. These criteria include both invasive and non-invasive measures of the hallmark histopathological lesions of SHI, namely inflammation, microvasculopathy and fibrosis [4]. The development of the criteria has intentionally targeted the measurement of these changes using investigation modalities currently applied in clinical practice.

Reflective of the diversity of potential SHI manifestations and the lack of a single pathognomonic feature of SHI [1], no single criterion is sufficient for a patient to be classified as having SHI. However, the criteria are not an exhaustive list of all cardiac abnormalities that can be attributed to SSc. The criteria have been developed with an intentional preference for item specificity over sensitivity, consistent with recommendations from the ACR [15]. For example, diastolic dysfunction is a common cardiac abnormality associated with SSc [16, 17], but there is overlap between echocardiographic parameters of healthy individuals and those with SHI [18]. Normal ageing is associated with impaired ventricular relaxation [18]. Therefore, a higher threshold of positivity, Grade II diastolic dysfunction, was established for this item to capture changes more specific to underlying pathology rather than potentially normal phenomena. Prioritizing specificity minimizes the risk of false-positive classification of patients and enables standardization of research practices, ensuring a consistent disease entity is being studied [15]. We acknowledge that prioritizing specificity will result in some patients with SHI being misclassified as not SHI. However, this trade-off was considered appropriate given the intended use of these criteria for research classification, with alternative lower thresholds potentially more suitable other contexts such as development of screening algorithms.

Use of CMR is becoming more routine and it is the only non-invasive technique able characterize myocardial inflammation and scar [19, 20]. CMR parametric mapping techniques can identify diffuse myocardial oedema and fibrosis. CMR has been proposed as a screening tool for SHI [21], and its use is likely to rapidly expand in the diagnosis of both sub-clinical and manifest cardiac disease in SSc. Endomyocardial biopsy (EMB) has utility in the diagnosis of SHI, however the procedure carries potential risks and may only be available at specialist centres. Additionally, it may not be routinely required with the increasing availability of CMR. Our study cohort reflected this with the infrequent use of EMB to secure a diagnosis of SHI. Furthermore, there is no universal histopathological criteria for diagnosis of SHI by EMB [22]. Our criteria make reference to the Dallas criteria [23], widely used histopathological criteria for the identification of myocarditis [22]. There are no routinely used pathological criteria to define excess fibrosis, with this defined as ‘fibrosis in excess of what would be expected for the patient’s age’. Qualitative descriptions of excess fibrosis are included in the descriptions and classification of other cardiomyopathies [24–26].

These classification criteria have explicit exclusion criteria. In the absence of a single diagnostic test or pathognomonic finding of SHI, there remains a requirement to exclude other causes of cardiac disease [8]. For example, comorbid PAH and SRC are exclusion criteria because of their known cardiac sequelae. It is not currently possible to distinguish between cardiac complications of PAH and SRC from SHI. As understanding of the genetic and molecular underpinnings of SHI improves, it may be possible to distinguish between primary and secondary cardiac consequences of SSc, potentially removing the need to have exclusion criteria as part of SHI classification criteria.

These criteria will not capture all patients who have SHI. As with other classification criteria, they are intended for use in research rather than to guide individual patient management. They have been deliberately designed to prioritize specificity, minimizing the false-positive classification of patients. Failure to meet the classification threshold should not preclude a diagnosis or treatment of SHI, which should be based on appropriate clinical assessment and investigation [15]. In particular, patients with a high clinical suspicion of SHI may be less likely to meet the classification threshold when advanced investigation such as CMR or EMB are not available. These criteria do not recommend that all cardiac investigations be performed in all SSc patients. Nor should the list of exclusion criteria be interpreted as a list of tests that must be obtained in all cases of suspected SHI.

This study has several strengths, including the use of expert consensus and decision analysis to derive and weight SHI criteria items. Input from experts in the fields of rheumatology, cardiology, cardiac imaging and anatomical pathology was sought. There was wide geographic representation from experts and test cases from across North and Central America, Europe, the UK and Asia-Pacific. This was intentional, to develop criteria that can be widely applied. The criteria were tested in a patient cohort developed without knowledge of the SHI classification criteria, minimizing circularity of reasoning in the testing of the criteria.

There are limitations of this study. No case had results for all cardiac investigations that can be used to identify SHI. The testing of these criteria was performed in a cohort reflective of real-world practice. It is likely that some cases rated as somewhat likely or uncertain by the submitting physician may have had a diagnosis of SHI secured or definitively excluded if additional investigations had been performed. When investigations were not performed, corresponding criteria were scored zero. This approach likely reduces the sensitivity of the final classification criteria as patients who do not have access to advanced imaging or invasive diagnostic testing services may be under-classified. This strategy was adopted to avoid assumptions about the presence of unmeasured pathology. As a result, missing data in the test cohort likely contributed to an underestimation of the sensitivity of the classification criteria. Florid SHI is an uncommon manifestation of a rare disease, with a prevalence of ∼5% in large SSc cohorts [27, 28]. Therefore, the generation of a combined cohort of 168 patients from multiple centres globally is one of the most significant efforts to collate detailed data about the various cardiac manifestations of SSc. Many cases were retrospective cases from contributing physicians’ clinical practice, meaning cases may be subject to the recall bias of contributing physicians. These criteria would benefit from prospective evaluation in larger cohorts that include greater numbers of patients with SHI. The small size of the test cohort mean validation study results should be considered preliminary pending further testing in larger cohorts. Prospective studies will allow standardized application of cardiac investigations across all participants, enabling more robust assessment of the sensitivity and specificity of the SHI classification criteria, and further testing of the criteria’s discriminatory performance.

Testing and validating classification criteria are inherently challenging without a definitive gold standard. At present, physician diagnosis is the gold standard for identification of SHI, with no minimum test requirement to secure the diagnosis [8]. This reference standard is subjective and prone to interobserver variability. Even amongst experts, we observed significant variation in the assessment of cases. This subjectivity has important implications for the performance characteristics of the proposed criteria. Sensitivity and specificity estimates are conditional on the reference standard used, and misclassification within the reference diagnosis may bias these estimates. Similarly, variability in physician assessment is likely to influence the optimal classification threshold. The identified threshold of ≥11 reflects expert clinical judgement and not an objective biological standard. Expert review of controversial cases to reach consensus as to the presence of SHI is a strength of this project as it mitigates, but does not eliminate, individual clinician bias. Nonetheless, the sensitivity and specificity of the final criteria may have been affected by variability in physician assessment. This limitation is inherent to the first iteration of any classification criteria and underscores the need for future refinement and validation in independent cohorts as objective imaging, histopathological and circulating biomarkers for SHI emerge. As methods of assessing cardiac inflammation, fibrosis and vasculopathy improve [29–33], these criteria will need to be revisited, with reconsideration of both item definition and individual item weights. The classification threshold required to identify cases of definite SHI may change as newer investigation modalities are implemented in clinical practice.

In conclusion, we present the first classification criteria for SHI. Using a combination of consensus and data-driven approaches, we have developed criteria that have good sensitivity and excellent specificity, and can identify SHI across the spectrum of disease. They represent a significant advance in this evolving field. We encourage their implementation in future studies to validate their use and to encourage investigation of this important disease manifestation.

## Supplementary Material

keag109_Supplementary_Data

## Data Availability

Data are available from corresponding author upon reasonable request.
