# Supplementary material for: Scleroderma clinical trials consortium classification criteria for systemic sclerosis heart involvement
Source: Rheumatology (Oxford). 2026 Feb 27;65(3):keag109. doi: 10.1093/rheumatology/keag109 (PMC13019150; doi:10.1093/rheumatology/keag109)
Supplement: keag109_Supplementary_Data [file keag109_supplementary_data.docx]

Supplementary material

*Supplementary Table S1: Summary of results physician assessments of test cases to determine classification threshold**

|  | **1** | **2** | **3** | **4** | **5** | **6** | **7** | **8** | **9** | **10** | **11** | **12** | **13** | **14** | **15** | **16** | **17** | **18** | **19** | **20** |
| --- | --- | --- | --- | --- | --- | --- | --- | --- | --- | --- | --- | --- | --- | --- | --- | --- | --- | --- | --- | --- |
| **Physician 1** | 20 | 11 | 11 | 10 | 20 | 12 | 15 | 10 | 19 | 12 | 13 | 19 | 11 | 19 | 16 | 18 | 5 | 12 | 4 | 8 |
| **Physician 2** | 16 | 16 | 9 | 10 | 16 | 10 | 9 | 10 | 17 | 10 | 9 | 14 | 1 | 17 | 12 | 10 | 9 | 6 | 12 | 16 |
| **Physician 3** | 18 | 10 | 10 | 10 | 18 | 15 | 15 | 15 | 12 | 18 | 18 | 10 | 4 | 15 | 15 | 12 | 6 | 15 | 15 | 15 |
| **Physician 4** | 20 | 15 | 4 | 3 | 13 | 3 | 12 | 3 | 7 | 10 | 11 | 5 | 13 | 16 | 15 | 10 | 5 | 8 | 9 | 17 |
| **Physician 5** | 17 | 15 | 15 | 5 | 15 | 10 | 15 | 13 | 13 | 10 | 13 | 10 | 0 | 10 | 10 | 10 | 10 | 5 | 10 | 15 |
| **Physician 6** | 18 | 8 | 14 | 3 | 16 | 7 | 16 | 5 | 12 | 6 | 7 | 8 | 2 | 17 | 15 | 7 | 3 | 3 | 8 | 13 |
| **Physician 7** | 19 | 15 | 16 | 10 | 10 | 10 | 10 | 10 | 12 | 8 | 12 | 14 | 10 | 18 | 15 | 15 | 4 | 10 | 15 | 10 |
| **Physician 8** | 20 | 9 | 13 | 12 | 17 | 3 | 13 | 8 | 13 | 10 | 15 | 10 | 0 | 20 | 18 | 7 | 6 | 13 | 4 | 5 |
| **Physician 9** | 19 | 17 | 6 | 7 | 19 | 8 | 16 | 7 | 7 | 8 | 10 | 7 | 0 | 17 | 11 | 13 | 6 | 10 | 15 | 4 |
| **Physician 10** | 20 | 17 | 10 | 10 | 15 | 10 | 10 | 7 | 5 | 5 | 17 | 15 | 2 | 20 | 13 | 15 | 5 | 14 | 10 | 20 |
| **Physician 11** | 19 | 18 | 10 | 16 | 10 | 4 | 15 | 5 | 12 | 2 | 15 | 10 | 12 | 19 | 16 | 18 | 2 | 4 | 2 | 15 |
| **Physician 12** | 18 | 17 | 15 | 7 | 18 | 15 | 17 | 12 | 3 | 7 | 15 | 7 | 0 | 16 | 15 | 14 | 7 | 15 | 7 | 17 |
| **Physician 13** | 20 | 15 | 10 | 10 | 19 | 10 | 10 | 10 | 18 | 10 | 16 | 13 | 10 | 18 | 19 | 18 | 10 | 10 | 10 | 11 |
| **Physician 14** | 10 | 13 | 6 | 10 | 14 | 8 | 14 | 10 | 11 | 7 | 4 | 13 | 1 | 18 | 15 | 17 | 6 | 10 | 5 | 7 |
| **Physician 15** | 18 | 12 | 11 | 9 | 13 | 8 | 10 | 8 | 11 | 6 | 9 | 17 | 7 | 17 | 11 | 11 | 4 | 8 | 8 | 8 |
| **Physician 16** | 19 | 17 | 12 | 6 | 2 | 8 | 13 | 10 | 16 | 2 | 4 | 16 | 5 | 16 | 15 | 15 | 4 | 10 | 11 | 13 |
| **Physician 17** | 18 | 13 | 13 | 10 | 6 | 7 | 15 | 10 | 8 | 10 | 10 | 15 | 3 | 12 | 12 | 15 | 5 | 10 | 13 | 14 |
| **Physician 18** | 20 | 13 | 12 | 12 | 18 | 13 | 16 | 10 | 15 | 12 | 14 | 13 | 5 | 20 | 15 | 20 | 6 | 12 | 12 | 12 |
| **Physician 19** | 20 | 18 | 0 | 1 | 17 | 2 | 6 | 1 | 18 | 0 | 3 | 2 | 0 | 4 | 4 | 10 | 2 | 4 | 3 | 19 |
| **Physician 20** | 20 | 20 | 14 | 4 | 1 | 16 | . | 10 | 20 | . | . | 20 | 0 | . | 18 | 18 | . | . | 8 | 20 |
| **Physician 21** | 18 | 15 | 12 | 10 | 17 | 10 | 16 | 7 | 16 | 4 | 12 | 12 | 3 | 18 | 12 | 17 | 3 | 12 | 10 | 19 |
| **Physician 22** | 18 | 14 | 10 | 7 | 18 | 8 | 18 | 7 | 7 | 10 | 6 | 12 | 2 | 16 | 15 | 7 | 10 | 6 | 13 | 18 |
| **Physician 23** | 20 | 16 | 9 | 5 | 12 | 12 | 12 | 4 | 11 | 3 | 13 | 12 | 10 | 18 | 18 | 19 | 6 | 14 | 15 | 13 |
| **Physician 24** | 18 | 12 | 10 | 10 | 14 | 11 | 16 | 16 | 15 | 16 | 15 | 10 | 16 | 17 | 11 | 17 | 15 | 18 | 10 | 20 |
| **Physician 25** | 20 | 5 | 6 | 10 | 15 | 13 | 18 | 14 | 15 | 13 | 10 | 10 | 2 | 17 | 18 | 17 | 0 | 15 | 14 | 20 |
| **Physician 26** | 18 | 14 | 2 | 0 | 2 | 15 | 0 | 16 | 15 | 3 | 4 | 4 | 4 | 16 | 14 | 16 | 1 | 1 | 3 | 6 |
| **Physician 27** | 19 | 15 | 10 | 13 | 20 | 11 | 20 | 14 | 8 | 11 | 17 | 15 | 12 | 19 | 14 | 18 | 4 | 15 | 10 | 19 |
| **Physician 28** | 17 | 13 | 12 | 8 | 16 | 10 | 10 | 10 | 13 | 6 | 12 | 13 | 8 | 15 | 15 | 17 | 5 | 8 | 10 | 18 |
|  |  |  |  |  |  |  |  |  |  |  |  |  |  |  |  |  |  |  |  |  |
| **SCTC Classification Score** | 28 | 19 | 7 | 4 | 18 | 12 | 19 | 12 | 11 | 16 | 11 | 14 | 0 | 18 | 7 | 21 | 0 | 9 | 8 | 31 |

*Cases ranked on scale of 0-20; 0 = definitely not systemic sclerosis heart involvement, 20 = definitely systemic sclerosis heart involvement. SCTC Classification Criteria score calculated by summing individual positive criteria scores.

*Supplementary Table S2: Sensitivity and specificity of tested classification thresholds for SHI*

| **Classification**  **Threshold** | **Sensitivity** | **Specificity** | **Percentage of patients correctly classified** | **Area under the curve** | **Odds Ratio** |
| --- | --- | --- | --- | --- | --- |
| **>8** | 84.44% | 91.87% | 89.88% | 0.88  (0.82-0.94) | 61.34  (21.82-172.42) |
| $\boldsymbol{\geq}$**11** | 77.78% | 95.93% | 91.07% | 0.87  (0.80-0.93) | 82.60  (26.47-257.72) |
| **>12** | 66.67% | 95.93% | 88.10% | 0.81  (0.74-0.88) | 47.20  (15.89-140.19) |
| **>14** | 57.78% | 97.56% | 86.90% | 0.78  (0.70-0.85) | 54.74  (15.08-198.72) |
